# Fetal Radiation Exposure Induces Testicular Cancer in Genetically Susceptible Mice

**DOI:** 10.1371/journal.pone.0032064

**Published:** 2012-02-13

**Authors:** Gunapala Shetty, Paul B. Comish, Connie C. Y. Weng, Angabin Matin, Marvin L. Meistrich

**Affiliations:** 1 Department of Experimental Radiation Oncology, The University of Texas MD Anderson Cancer Center, Houston, Texas, United States of America; 2 Department of Genetics, The University of Texas MD Anderson Cancer Center, Houston, Texas, United States of America; Baylor College of Medicine, United States of America

## Abstract

The prevalence of testicular germ cell tumors (TGCT), a common solid tissue malignancy in young men, has been annually increasing at an alarming rate of 3%. Since the majority of testicular cancers are derived from germ cells at the stage of transformation of primordial germ cell (PGC) into gonocytes, the increase has been attributed to maternal/fetal exposures to environmental factors. We examined the effects of an estrogen (diethylstilbestrol, DES), an antiandrogen (flutamide), or radiation on the incidence of testicular germ cell tumors in genetically predisposed 129.MOLF-L1 (L1) congenic mice by exposing them to these agents on days 10.5 and 11.5 of pregnancy. Neither flutamide nor DES produced noticeable increases in testis cancer incidence at 4 weeks of age. In contrast, two doses of 0.8-Gy radiation increased the incidence of TGCT from 45% to 100% in the offspring. The percentage of mice with bilateral tumors, weights of testes with TGCT, and the percentage of tumors that were clearly teratomas were higher in the irradiated mice than in controls, indicating that irradiation induced more aggressive tumors and/or more foci of initiation sites in each testis. This radiation dose did not disrupt spermatogenesis, which was qualitatively normal in tumor-free testes although they were reduced in size. This is the first proof of induction of testicular cancer by an environmental agent and suggests that the male fetus of women exposed to radiation at about 5–6 weeks of pregnancy might have an increased risk of developing testicular cancer. Furthermore, it provides a novel tool for studying the molecular and cellular events of testicular cancer pathogenesis.

## Introduction

Testicular germ cell tumors (TGCT) are the most common malignant tumor in Caucasian men aged between 15 and 40 years. Human TGCTs are mainly classified histologically into seminomas, which resemble the primordial germ cells (PGCs), and non-seminomas, which are either undifferentiated (embryonal carcinoma) or differentiated showing embryonic (teratoma) or extra-embryonic (yolk sac) patterning [1]. Teratomas are characterized by the differentiation to a diverse array of cell and tissue types. The median age of onset of various tumor types differs with seminomas arising at about 35 years, most non-seminomas at 25 years, and most teratomas and yolk sac tumors at around 1.5 years. Whereas the tumors appearing in adults are preceded by carcinoma in situ (CIS), which contain cells closely resembling gonocytes, those appearing in children are not preceded by CIS [2]. In spite of these differences evidence supports a common underlying pathogenesis of these tumors such as similar chromosome aberrations, amplified chromosomal regions, and expression of pluripotency genes [3]. All of these tumors are derived from cells with characteristics of primordial germ cells (PGC) or gonocytes, although at different stages of development assessed by the extent of erasure of the original biparental imprinting or the later establishment of paternal imprinting.

There has been an annual increase of 3% in the incidence of TGCTs in young Caucasian men throughout the world in the past 50 years [4], but the reasons are elusive. Elucidation of the cause of this increase is important for possible prevention or reversal of this increase.

Since testicular cancer cells are derived from primordial germ cells (PGCs) or gonocytes [2] the increase has been attributed to maternal and fetal exposures to environmental factors, with most attention given to endocrine disruptors such as estrogens and antiandrogens [5], [6], [7]. Ionizing radiation, a known carcinogen [8], that increases the incidence of childhood and other adult cancers in individuals exposed during fetal development [9], [10], has received little study. One case-control study did show that exposure to ionizing radiation during pregnancy increased the risk of testicular cancer in male offspring [11].

The only murine model for TGCT is the spontaneous teratomas that were originally observed in 129/Sv mice [12]. They appear most similar to the infantile TGCTs in human because germ cells rapidly develop into tumors after birth. However, like all human TGCT types, the mouse teratomas also originate from the PGCs or gonocytes. In addition, defects in the same genes, kit-ligand (KITL) and Dmrt1, dramatically increase the incidence of teratoma in mice [13], [14] and are associated with or predispose humans to the adult TGCTs [15], [16]. These examples suggest that this mouse teratoma model may be relevant to the adult forms of human testicular cancer as well, although there are reservations about such an extrapolation [1].

In the 129/Sv mice, about 3–10% develop spontaneous TGCTs. Congenic mice on a 129 background have been developed; those containing all of chromosome 19 derived from the MOLF strain have an 80% incidence of TGCTs [17] and those carrying portions of chromosome 19 display varying levels of tumor incidence [18], [19]. In the current study, we employed the 129.MOLF-L1 (L1) congenic strain, with a reported 30% incidence of TGCTs, to maximize the power of the study. We used this mouse model to test the effect of estrogen, antiandrogen and ionizing radiation exposure on TGCT incidence. Exposures were done at E10.5 and 11.5 which is just after the primordial germ cells (PGCs) colonize the fetal gonad, and are undergoing extensive epigenetic changes that suppress their potential to revert to a pluripotent state and commit them to germ cell development [20].

## Results

We examined the effects of treatments with an estrogen (diethylstilbestrol, DES), an antiandrogen (flutamide), or irradiation on the incidence of TGCT in an animal model. We exposed pregnant L1 females to the agents at days 10.5 and 11.5 of pregnancy.

There was no effect of flutamide, DES or irradiation on the reproductive ability of the dams (Table 1). This was confirmed by the similar percentages of mice producing progeny after successful mating and the comparable litter sizes in the treated and respective control dams.

**Figure 1:**
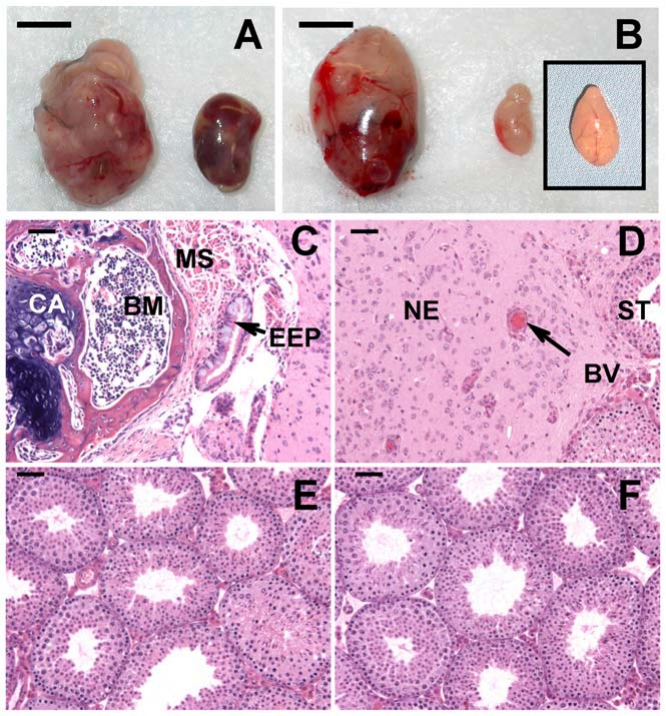
Morphology and histology of the testes in 4-week-old 129.MOLF-L1 male mice irradiated *in utero* with two doses of 0.8 Gy and controls. Testicular morphology showing bilateral (**A**) and unilateral (**B**) TGCTs from irradiated mice. Note that the testis without TGCT after *in utero* radiation exposure (B) is smaller than the normal untreated testis from a 4-week-old mouse (insert in B). Testis sections showing teratoma containing tissues apparently from multiple dermal origins identified by morphology (**C**), and TGCT containing only neuroepithelial cells (NE) (**D**). CA: cartilage; BM: bone marrow; MS: muscle, EEP: endodermal epithelium. ST: seminiferous tubule; BV: blood vessel. Sections from testes of 4-week old irradiated (**E**) and contol (**F**) mice without TGCT showing qualitatively normal spermatogenesis. Bar: 0.5 cm in A & B, and 50 µm in C–F.

Testes were harvested from the male offspring and many of them contained visible tumors (Figure 1A and B). Histological examination confirmed that most of these were teratomas with tissues from multiple dermal origins, but some contained only neuroepithelial cells (Figure 1C and D). Flutamide did not significantly increase the incidence of total TGCTs (30% of testes had tumors vs. 21% in control). However, it did slightly increase the incidence of TGCTs that were confirmed to be teratomas by the presence of multiple dermal origin cell types (24% of testes vs. 14% in control, *P* = 0.04) (Table 2).

**Table 1:** Breeding efficiency in 129.MOLF-L1 congenic mice with or without *in utero* flutamide, DES, or radiation treatment.

Treatment	Plug positive	Produced progeny^a^	Litter size^b^
Control	48	19 (40%)	4.9±0.3
Flutamide	36	23 (64%)	5.1±0.6
Control	5	3 (60%)	4.6±0.3
DES	12	6 (50%)	4.5±0.7
Control	ND	ND	6.4±0.7
Radiation	28	15 (54%)	5.3±0.6

a Values given as absolute number and percentage of total plug-positive females.

b Mean ± SEM.

**Table 2:** Testicular germ cell tumor (TGCT) incidence and related characterization in 129.MOLF-L1 congenic mice with or without *in utero* flutamide, DES, or radiation treatment.

Treatment	No. males analyzed	Overall TGCT^b^ per mouse	Bilateral TGCT^b^ per mouse	No. testes analyzed	TGCT^b^ per testis	% identified as teratomas^b^	Weight (mg)^a^ testes with no TGCT	Weight (mg)^a^ testes with TGCT
Control	54	21 (39%)	2 (4%)	108	23 (21%)	15 (65%)	50±1 (n = 84)	96±14 (n = 22)
Flutamide	51	24 (47%)	7 (14%)	102	31 (30%)	27 (87%)	49±1 (n = 55)	109±18 (n = 25)
Control	5^c^	1 (20%)	0 (0%)	10	1 (10%)	0 (0%)	46±2 (n = 9)	58 (n = 1)
DES	12	2 (17%)	1 (8%)	24	3 (13%)	3 (100%)	47±2^d^ (n = 19)	161±93 (n = 3)
Control	20	9 (45%)	2 (10%)	40	11 (28%)	7 (64%)	60±1 (n = 30)	76±9 (n = 10)
Radiation	23	23 (100%)^e^	14 (61%)^e^	46	37 (80%)^e^	35 (95%)^e^	36±1^f^ (n = 9)	158±28^f^ (n = 37)

a Mean ± SEM.

b Values given as absolute number and percentage of mice, testes, or tumors analyzed.

c Since the incidence of tumors in the DES-treated mice was not increased from historical controls or the concurrent flutamide controls, we did not complete this arm of the study, and cannot rigorously conclude that there is no increase in tumor incidence in DES-treated mice compared to a sham-treated control group.

d Two cryptorchid testes (weights, 19 and 34 mg) were excluded from this average.

e Significantly different between treated and control mice, Fisher's exact Chi square test: *P*<0.01. Other differences were not significant (*P*≥0.05).

f Significantly different between treated and control mice, *t* test: *P*<0.01. Other differences were not significant (*P*≥0.05).

The dose of DES given was effective in acting on the embryo as it caused cryptorchidism in 16% of the testes. However, there was no indication that it induced cancer (only 3 of 24 testes analyzed contained TGCTs). Similarly in a study involving treatment of pregnant 129 mice, a dose of ethinyl estradiol that increased cryptorchidism in the offspring did not induce a significant change in the incidence of teratomas [21].

In contrast, irradiation of the males during fetal development with two doses, each of 0.8 Gy, led to TGCTs in 80% of the testes (compared to 28% in control) and in 100% of the male offspring (Table 2). The numbers of mice with unilateral and bilateral tumors in all groups were binomially distributed, confirming a previous report that the occurrence of a tumor in each testis was an independent event [18]. The weights of testes with TGCTs and the percentage of tumors that were confirmed teratomas were higher in the irradiated mice than in controls, indicating that irradiation induced more aggressive tumors and/or more foci of initiation sites in each testis.

The two radiation doses of 0.8 Gy did not disrupt spermatogenesis, which was qualitatively normal in testes that did not contain teratoma (Figure 1E and F). However, these tumor-free testes from irradiated mice had reduced weights compared to those from controls (Table 2), suggesting that irradiation had caused some loss or inhibition of proliferation of PGCs and/or somatic cells.

## Discussion

In the present study we unequivocally demonstrated that fetal radiation exposure during E10.5–E11.5 induces testicular germ cell cancers, most of which show the multiple dermal origins typical of teratomas, in a genetically susceptible mouse model. This is the first demonstration of induction of testicular cancer by an environmental agent when exposed during embryonic period. Further characterization of the model to determine the optimal exposure time or intervals, the dose-response, and the effects on less susceptible mouse strains is needed to initiate mechanistic studies and risk estimation.

The finding that radiation dramatically increased the incidence of testicular cancer in mice offers a new tool for investigating the mechanism by which the PGCs revert to pluripotent embryonic cells. When mouse PGCs arrive in the gonad at E10.5, they are potentially pluripotent. However, by day 15.5, they are gonocytes and have suppressed expression of pluripotency genes (*Oct4, Nanog*, *Sox2*), undergone cell cycle arrest, are expressing germ cell genes (*Nanos3, Dazl, Dnd1, Mvh*) [22], [23], and are committed to the germ cell self-renewal and differentiation pathway [12]. Between days E10.5 & 11.5 they undergo epigenetic changes that include both global DNA demethylation which involves single-strand breaks (SSBs) and activation of the base-excision repair pathway with chromatin localization of the proteins involved, as well as chromatin remodeling involving loss and replacement of histones and changes in their modifications [24]. Ionizing radiation itself induces larger numbers of SSBs and produces damaged bases. This radiation-induced damage might perturb signaling pathways involving endogenous SSBs and base-excision repair regulating this epigenetic transformation and allow the cells to retain pluripotency as embryonic stem-like cells that subsequently form the TGCTs.

Previously genetic alterations in germ cells, including the *ter* mutation in the *Dnd1* gene [25], loss of *Dmrt1* [14], or loss of *Pten* [26] produced a high incidence of TGCTs (particularly teratomas) in mice. Analysis of the changes in expression of these genes or gene products in PGCs and gonocytes of embryos of irradiated mice could elucidate the mechanism by which radiation acts to induce the tumors.

Although the extrapolation of the teratomas in mice to the sporadic testicular tumors in young adult human males is still debatable, the present results on *in utero* irradiation of mice suggest that the male fetus of women exposed to radiation at about 5–6 weeks of pregnancy might be at an increased risk of developing testicular cancer. Cohort studies are unlikely to be able to test whether there is an association between such exposure and testis cancer since X-ray doses in diagnostic procedures have been declining [9], the numbers of pregnant women in populations exposed to radiation are small [10], there is likely a limited developmental window of sensitivity, the incidence of testis cancer in human is low, and several decades elapse between exposure and appearance of tumors. Nevertheless, there is one report of an association between maternal ionizing radiation exposure during pregnancy and testicular cancer in their offspring [11]. The knowledge gained about mechanisms of ionizing radiation-induced testicular cancer in mice can be used to identify other environmental or lifestyle factors that might cause similar damage to fetal germ cells and be responsible for the current increases in testicular cancer incidence in men.

## Materials and Methods

### Mice and breeding

A recently described inbred, congenic mouse strain, 129.MOLF-L1 [19], referred to as L1 was used. These were originated from crosses between the 129 (129S1/SvImJ) strain and MOLF/Ei inbred mice of the *Mus m. molossinus* mouse subspecies. To create L1 mice 129 consomic mice with MOLF-Chr19 mice were backcrossed to 129, selecting for a 7.6 Mb region from the MOLF chromosome 19, and then made homozygous for MOLF-derived region by intercrossing the progeny. About 30% of the L1 males were reported to develop spontaneous testicular tumors and the MOLF genes contributing to this high tumor incidence are not yet known [19].

Timed matings were performed with pairs of L1 mice. Pregnant females on days 10.5 and 11.5 of their pregnancy were treated with two daily doses of the chemical or physical agent as described below. In some cases females were used for a second or third round of treatments during consecutive pregnancies with a waiting period of a few weeks between pregnancies. In such cases of multiple treatments, pregnant females received either the same agent or they received control treatment in each pregnancy.

All experimental procedures were approved by the MD Anderson Cancer Center Institutional Animal Care and Use Committee with approved protocol numbers 110712631 and 110712632. All facilities for housing animals are registered by the USDA and accredited by the American Association for the Accreditation of Laboratory Animal Care.

### Treatments

#### Flutamide

Flutamide (Sigma Aldrich, St. Louis, MO), dissolved in sesame oil/absolute ethanol (1∶1), was given subcutaneously as 2 doses of 40 mg/kg. This dose has been shown to block androgen action in male mice [27] and is higher than the human daily clinical oral dose of ∼10 mg/kg.

#### Diethylstilbestrol (DES)

DES (Sigma Aldrich, St. Louis, MO), dissolved in corn oil, was given subcutaneously as two doses of 5 µg/kg. Control pregnant mice received only the vehicle. Based on a previous report [28], we performed initial experiments with two doses of 20 µg/kg but reduced the dose after many of the pregnant females failed to deliver offspring.

#### Radiation

Pregnant female L1 mice were irradiated with Co^60^-gamma radiation (Eldorado 8 radiation unit, Atomic Energy of Canada Ltd., Ottawa, Canada). A maximum of 3 unanesthetized mice at a time were placed in a ventilated methacrylate box (4.1 cm×11.7 cm×8.2 cm) on a base of a minimum of 13 cm of pressed wood, at a distance of 80 cm from the radiation source. With a field size of 20×20 cm, a dose of 0.8 Gy whole body radiation was administered at 10–11 AM on days 10.5 and 11.5 of pregnancy at a dose rate of about 70 cGy/min. The control L1 dams from the same breeding colony did not receive any of these treatments or manipulations and the male offspring from these mice were analyzed concurrently with those of the treated ones.

### Analysis

The *in utero* exposed and control male offspring in the flutamide arm were euthanized at the age of 4–5 weeks; the exposed and control males in the radiation and DES arms were euthanized at the age of 4 weeks. This time was chosen since tumors in L1 mice are visually observed at these ages [19]. The testes were examined for the presence of tumors by visual observation and then fixed in Bouin's solution for histologic examination. As some small tumors were not evident by visual examination all tumors were identified by the analysis of hematoxylin and eosin-stained 5-µm sections. Tumors with tissues from multiple dermal types were recognized as teratomas. Whenever, the initial testis section contained only cancerous neuroepithelial cells, the blocks were re-sectioned to identify or rule out the presence of other cancerous tissue types in other locations.

### Statistical Analysis

The data are presented as mean ±SEM. The significance of differences between the testis weights from mice of the same age was evaluated by a Student's *t*-test. The significance of the differences in the frequency of TGCTs between treated and control mice was determined using the Chi-square test.
